# A pilot randomised controlled trial of a web-based implementation intervention to increase child intake of fruit and vegetables within childcare centres

**DOI:** 10.1186/s40814-020-00707-w

**Published:** 2020-10-29

**Authors:** Courtney Barnes, Alice Grady, Nicole Nathan, Luke Wolfenden, Nicole Pond, Tameka McFayden, Dianne S. Ward, Amber E. Vaughn, Sze Lin Yoong

**Affiliations:** 1 Hunter New England Population Health, Locked Bag 10, Wallsend, NSW 2287 Australia; 2 School of Medicine and Public Health, The University of Newcastle, Callaghan, NSW Australia; 3 Hunter Medical Research Institute, New Lambton, Australia; 4 Priority Research Centre for Health Behaviour, The University of Newcastle, Callaghan, Australia; 5 Department of Nutrition, Gillings School of Global Public Health, University of North Carolina, Chapel Hill, NC USA; 6 Center for Health Promotion and Disease Prevention, University of North Carolina, Chapel Hill, NC USA

**Keywords:** Child diet, Obesity, Childcare centre, Web-based, Implementation, Randomised controlled trial, Intervention

## Abstract

**Background:**

As dietary behaviours developed during early childhood are known to track into adulthood, interventions that aim to improve child nutrition at a population level are recommended. Whilst early childhood education and care (ECEC) is a promising setting for interventions targeting children’s nutrition behaviours, previous interventions have largely used high intensity, face-to-face approaches, limiting their reach, implementation and potential impact at a population level. Web-based modalities represent a promising means of supporting the delivery of childcare-based interventions whilst overcoming challenges of previous approaches; however, the feasibility of using such modalities to support implementation is largely unknown. As such, this study sought to collect feasibility and pilot data to inform the design of a web-based intervention together with health promotion officer support within childcare centres. Child dietary intake will also be assessed to provide an estimate of the impact of the implementation intervention.

**Methods:**

A superiority cluster randomised controlled trial with repeat cross-sectional data collection employing an effectiveness-implementation type-II hybrid design will be conducted with childcare centres within the Hunter New England region of New South Wales, Australia. Type-II hybrid designs provide the opportunity to assess intervention efficacy whilst piloting the feasibility of the implementation strategies. Centres allocated to the intervention group will receive access to a web-based program together with health promotion officer support to implement targeted healthy eating practices to improve child diet in care. A number of outcomes will be assessed to inform the feasibility to conduct a larger trial, including childcare centre and parent recruitment and consent rates for each component of data collection, uptake of the implementation strategies, acceptability of the intervention and implementation strategies, appropriateness of the implementation strategies and the contextual factors influencing implementation.

**Discussion:**

This study will provide high-quality evidence regarding the potential feasibility of a web-based intervention and the impact of healthy eating practices on child diet in care. Web-based modalities provide a promising approach for population-wide implementation support to childcare centres given their potential reach and consistency with existing infrastructure.

**Trial registration:**

Prospectively registered with Australian New Zealand Clinical Trial Registry (ACTRN12619001158156).

**Supplementary Information:**

**Supplementary information** accompanies this paper at 10.1186/s40814-020-00707-w.

## Background

Childhood overweight and obesity increases the risk of adult obesity and several other chronic diseases, including cardiovascular disease, type 2 diabetes and specific cancers [1]. Internationally, more than 41 million children aged 0–5 years were classified as overweight or obese in 2016 [1]. Poor dietary behaviours, including a low intake of fruit and vegetables and a high intake of energy-dense discretionary food and beverages (those which are high in sodium, saturated fat and added sugars), are considered to be primary risk factors for the development of childhood overweight and obesity [2]. Current evidence from Australia, the United States (U.S.) and the United Kingdom (U.K.) report that over 90% of pre-school aged children do not consume recommended servings of vegetables, and almost all consume excessive amounts of discretionary foods [3–5]. Although substantially higher than vegetables, evidence suggests that an inadequate proportion of children are consuming the recommended servings of fruit [6]. As dietary behaviours developed during early childhood are known to track into adulthood [7], interventions that aim to improve child nutrition at a population level are recommended [8].

Early childhood education and care (ECEC) is a promising setting for interventions targeting children’s nutrition behaviours. With at least 80% of children in countries including Australia and the U.K. attending formal centre-based childcare (herein referred to as childcare centres) [4, 9], including long day care and preschools, interventions targeting this setting have the potential to reach a large number of children during a crucial developmental period. In Australia, children attend care for an average of 21 h per week [10], providing multiple opportunities to reinforce healthy eating behaviours. Furthermore, as children can consume up to two thirds of their recommended daily intake whilst in care [11], interventions to improve the healthy eating environments of childcare centres have the potential to substantially improve a child’s overall nutrition intake. Internationally, sector-specific recommendations for childcare centres exist [12–14] which acknowledge the potential impact of the childcare centre environment on influencing children’s dietary intake. Importantly, two recent scoping reviews summarising findings from systematic reviews have identified several childcare-based nutrition practices associated with improved child diet outcomes. These include the provision of interactive child education to improve child knowledge and skills, educator positive role modelling and engaging with parents to target the provision of healthier foods [15, 16].

Whilst the implementation of such practices could potentially improve child nutrition, studies show that such practices are not routinely implemented by childcare staff [17]. A 2016 systematic review of implementation support strategies in the childcare setting suggests that comprehensive approaches, addressing multiple barriers to implementation, may be most effective in improving centre uptake of evidence-based nutrition interventions and dietary intake of children in care [18]. However, such approaches are often resource-intensive, relying on face-to-face training and ongoing support to childcare centre staff, which pose significant financial constraints to achieve sustained implementation at scale [18].

Web-based modalities may be a promising way of supporting the implementation of childcare-based interventions whilst overcoming some of the challenges of previous approaches. As 100% of childcare centres in Australia have access to and use a computer daily for reporting requirements [19], web-based interventions are likely to have a broader reach compared to traditional face-to-face modalities [19]. Additionally, web-based approaches have the potential to be more cost-effective than traditional approaches by reducing the burden on resources such as time and staffing and allowing access to the intervention at a time and place convenient to staff [15]. Further, behavioural strategies can be embedded within web-based interventions to deliberately target reported barriers to the implementation of healthy eating practices [20].

To our knowledge, only two randomised controlled trials (RCT) on the impact of a web-based program in childcare centres have been published. A pilot implementation RCT with 31 centres who provided food in care from within the U.S. assessed the impact of the web-based Nutrition and Physical Activity in Child Care (Go-NAPSACC) program on childcare healthy eating environments [20]. The study assessed improvements in childcare nutrition environments via childcare centre director self-report and found that centres who had received the intervention had improved self-reported nutrition environment scores [20]. Within Australia, one RCT with 54 centres evaluated the impact of a web-based menu planning program on centre provision of foods in accordance with sector dietary guidelines. Whilst the intervention did not result in a statistically significant increase in dietary guideline compliance, significant improvements in the provision and consumption of healthy foods and a significant decrease in unhealthy healthy foods were found [21, 22]. Whilst these studies show promise, little is known about the feasibility of implementing such an intervention within Australian childcare centres, particularly amongst those that are not responsible for providing food to children.

Given the limited existing evidence base, the primary objective of this study is to examine via a type-II hybrid cluster RCT, the potential feasibility of a web-based intervention together with health promotion officer support within childcare centres, whilst assessing the uptake, acceptability and appropriateness of the intervention and implementation strategies, and contextual factors influencing implementation.

Secondary objectives are to:

1. Examine the potential effects of the implementation strategy employed in the study on childcare centre implementation of recommended practices;
2. Assess the effectiveness of the intervention in increasing child dietary intake of fruit and vegetable servings, and decreasing child dietary intake of sodium (milligrams (mg)), saturated fat (grams (g)) and added sugar (grams (g)) in care; and
3. Assess the effectiveness of the intervention in increasing servings of fruit and vegetables packed within children’s lunchboxes.

## Methods and analysis

### Study design and setting

A superiority cluster RCT with repeat cross-sectional data collection employing an effectiveness-implementation type-II hybrid design will be conducted [23]. A hybrid effectiveness-implementation design enables the assessment of the feasibility of the intervention and the potential effects of an implementation strategy on centre implementation of healthy eating practices, whilst assessing the effectiveness of the intervention in improving child dietary intake of fruit and vegetables [24]. The study will take place in the Hunter New England (HNE) region of the state of New South Wales (NSW), Australia. The HNE region has approximately 422 centre-based childcare centres, including preschools and long day care, which typically enrol children aged 0–6 years for an average 21 h per week [10, 25]. The protocol is reported according to the Standard Protocol Items: Recommendations for Interventional Trials (SPIRIT) (Additional file 1) [26].

### Study population and recruitment

#### Childcare centres

To be eligible, childcare centres must (1) enrol > 20 children per day, (2) have internet access at the centre, (3) not provide meals or snacks to children (i.e. parents or caregivers must be required to provide food packed in lunchboxes), (4) not be currently participating in any other intervention to improve child healthy eating and/or physical activity and (5) not be fully compliant with healthy eating practices targeted by the intervention and specified in the NSW state obesity prevention program (i.e. *Munch & Move*) [27]. Mobile preschool, family day care centres and centres that do not cater to children aged 2–5 years, cater exclusively for children requiring specialist care, or are run by the Department of Education and Communities Centre will be excluded due to differing operational characteristics.

A list of potentially eligible centre-based childcare centres located within the HNE region will be provided by the NSW Ministry of Health [28]. Evidence-based recruitment strategies in the childcare setting will be employed to reduce risk of recruitment bias and maximise centre participation in the study [29–31]. Specifically, one member of the research team will coordinate centre recruitment and monitor consent rates [32]. A recruitment package consisting of a study information statement and consent form will be progressively distributed to potentially eligible centres in random order. Approximately 2 weeks later, a research assistant (RA) will telephone centres in random order to assess eligibility, review study details and request consent for study participation. Centres will continue to be contacted until the required number have consented. Such recruitment strategies have been used previously by the research team to obtain consent rates over 70% [33]. The RA will also schedule a 2-day site visit to complete baseline data collection for consenting centres. Centre-level information provided by the NSW Ministry of Health and demographic information collected during centre recruitment calls will be used to characterise non-participants and assess the potential for selection bias. To minimise attrition, centres will be contacted prior to follow-up data collection to thank them for their participation and to schedule a date for data collection at a time convenient to them [32, 34].

#### Children

In order for children to be eligible to participate, they must (1) have prior written consent from a parent or guardian, (2) be between the ages of 2 and 5 years and (3) not have a dietary restriction that requires specialised tailoring of their diet (e.g. allergies, intellectual or physical disability).

Approximately 2 weeks prior to data collection, centres will be asked to distribute parent information statements and consent forms via electronic methods, including email and parent communication apps, and child pigeonholes as part of standard communication with parents. The dates of the scheduled site visits will not be disclosed to parents to avoid any changes to parent usual lunchbox packing. Additionally, approximately 1 week prior to the scheduled site visit, and on the day of the scheduled site visits, two RAs trained in recruitment and data collection procedures will be present at the childcare centre to request written consent from parents for child participation in the study.

### Randomisation and blinding

Childcare centres will be randomly allocated following a block randomisation procedure in a 1:1 ratio to either intervention or control using a computerised random number function in Microsoft Excel 2013. Due to the demographic and socioeconomic diversity of the HNE region, randomisation will be stratified by centres with a high number of Aboriginal child enrolments (> 10%) and by centre socioeconomic status (SES), as determined by Socio-Economic Indexes for Areas categorisation using centre postcodes [35, 36]. Randomisation will be completed following baseline data collection by a statistician not otherwise involved in the trial. Staff at participating childcare centres and those delivering the intervention will be aware of group allocation. Every effort will be made to keep data collectors and analysts blind to group allocation. However, there is potential for data collectors to become aware of group allocation due to the nature of the intervention (e.g. display of intervention resources within the centre).

### Intervention practices targeted by the web-intervention

The intervention will target nominated supervisors and staff within childcare centres and support their implementation of five healthy eating practices. The selection of the targeted practices is broadly consistent with the social ecological framework (SEF) which posits that individual behaviour can be influenced via factors through five nested, hierarchical levels (individual, interpersonal, community, organisation and policy/enabling environment) [37]. Whilst the framework acknowledges that broader level factors influence behaviour, this intervention seeks primarily to influence child diet whilst attending childcare and, as such, primarily targets the individual and organisational determinants. The selection of the targeted practices are based on empirical evidence supporting the association between these practices and improved child dietary intake in childcare, or more generally in other settings [38, 39] as well as recommendations by international, national and state guidelines [13, 14, 27]

Specifically, childcare centres will be asked to implement the following targeted healthy eating practices within the 6-month intervention period:

i.) Supporting families to provide healthier foods consistent with dietary guidelines: Childcare centres will be asked to monitor children’s lunchboxes for consistency with dietary guidelines on a daily basis and distribute nutrition-focused messages to parents that promote the packing of healthy lunchboxes at least twice during the intervention period. Messages will offer advice to address parents’ commonly reported barriers to providing healthy foods, including overcoming fussy eating, improving food acceptance, providing healthy foods on a budget and quick and healthy options [36].
ii.) Provision of intentional learning experiences about healthy eating to children: Childcare centre staff will be asked to provide children with intentional learning experiences at least twice per week aimed to support children’s development of healthy eating behaviours [40]. Intentional learning experiences include, but not limited to, tasting sessions with new food, planting seeds within a vegetable garden and reading books about healthy foods.
iii.) Use of feeding practices that support children’s healthy eating: Childcare centre staff will be asked to provide positive reinforcement and encouragement to children to promote healthy eating and trying new foods at every meal and snack occasion. They will also be asked to model healthy food and drink choices at every meal, provide positive comments about healthy foods within children’s lunchboxes and avoid the use of food incentives to encourage desired behaviour [14, 15, 41].
iv.) Staff participation in professional development in healthy eating: Childcare centres will be asked to have at least 50% of staff take part in online training opportunities targeting staff healthy behaviours and practices in the centre [27, 42]. This training contains videos, interactive activities and reflective practice questions that will provide educators with the knowledge, skills and resources to embed healthy eating into their centre.
v.) Having a comprehensive written nutrition policy that outlines key healthy eating practices: Childcare centres will be asked to develop or modify their existing nutrition policy to ensure the centre has strategies, procedures and guidelines to enforce the implementation of healthy eating practices to improve child diet [43]. Childcare centres will be asked to include the following elements within the policy: strategies are in place to ensure staff monitor children’s lunchboxes daily for alignment with dietary guidelines, communication with families regarding foods packed within lunchboxes at least twice every 6 months, scheduling and delivery of intentional nutritional learning experiences at least twice per week, staff role modelling positive feeding practices at every meal and snack time to support children’s healthy eating and at least 50% of staff participate in professional development in healthy eating.

### Implementation

The Behavioural Change Wheel (BCW) [44] was used to identify specific components within the web-based program as well as other implementation support strategies that could be employed to support childcare staff to change their behaviour or their organisation to create supportive environments for child healthy eating, and therefore, potentially improve child diet intake in care [44]. Specifically, barriers and enablers to childcare staff behaviour change were identified through a systematic review of the literature [17, 45–48] and consultation with stakeholders, including childcare centre staff and health promotion officers (HPO) with experience working within the setting. The BCW process outlined by Michie et al. was then followed to categorise these barriers and enablers using the COM-B model as either capability, motivation or opportunity [44]. A summary of this process, including the behavioural change techniques (BCTs) employed within the intervention to address the barriers and enablers, is described in Table 1 [44]. The implementation support strategies, defined according to the Expert Recommendations for Implementing Change (ERIC) taxonomy [49], have been previously used by the research team within childcare-based interventions and aim to address reported barriers to intervention implementation whilst being embedded within current infrastructure of the units health promotion team [17, 50].

**Table 1:**
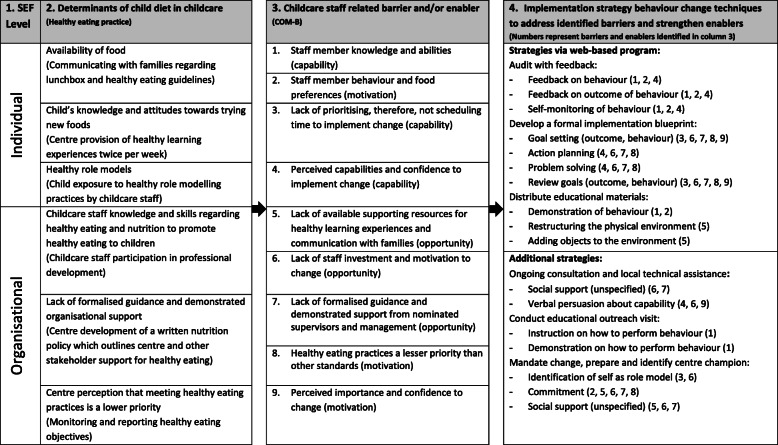
Determinants of child diet in childcare and strategies to address targeted barriers and enablers

Specifically, the implementation strategies incorporated into the web-based program, known as Childcare Electronic Assessment Tool and Support (EATS), include:

#### Audit and feedback

Childcare EATS includes a self-assessment of the implementation of targeted healthy eating practices. Following the completion of the self-assessment, the web-based program will immediately provide centres with feedback on practice performance. Childcare centres will be encouraged to complete the self-assessment at least twice during the intervention period to self-monitor improvements in practice [20, 33].

#### Develop a formal implementation blueprint

Following the completion of the self-assessment, childcare centres will be encouraged to use Childcare EATS to set goals and create an action plan to facilitate improvements in practice [20]. Centres will be encouraged to develop an action plan at least twice within the intervention period and continually monitor progress in consultation with centre staff to assist improvement in practice.

#### Distribute educational materials

Childcare EATS will house relevant materials developed through consultation with key stakeholders, including childcare centre staff, cultural liaisons and health promotion officers with extensive experience working within the setting. Materials were designed to assist centre’s adoption of targeted practices and include factsheets, email messages and newsletter snippets to facilitate communication with parents regarding children’s lunchbox alignment with guidelines; educational materials to improve staff knowledge of providing a positive healthy eating environment; example activities to demonstrate intentional nutrition learning experiences within the centre; directions to online learning opportunities, including webinars and eLearning modules to support staff professional development in nutrition; and nutrition policy templates [27].

In addition to web-based resources, childcare centres allocated to the intervention will receive support from HPOs within the local health district with experience working with childcare centres. The implementation strategies provided through these HPOs will include:

#### Identify and prepare a centre champion

Upon notification of group allocation, the HPO will ask centres to identify and prepare a staff member from the centre who will dedicate themselves to supporting, marketing and driving implementation of the intervention [49, 51].

#### Conduct educational outreach visit

Centre staff (nominated supervisor and centre champion) will receive one face-to-face training session by an HPO to support implementation of the healthy eating practices and introduce the web-based program at the beginning of the intervention period [35]. This will be a practical, hands-on training session to ensure staff are comfortable using Childcare EATS, accessing supporting resources and are aware of the key practices targeted by the intervention.

#### Mandate change

Centre nominated supervisors will be asked to show support for implementing targeted healthy eating practices via a memorandum of understanding, which will outline the responsibilities and expected commitment from both the childcare centre and HPO in working to improve the implementation of healthy eating practices to improve child dietary intake in care. The memorandum of understanding will be discussed and agreed upon during the educational outreach visit with centre supervisors.

#### Provide ongoing consultation and local technical assistance

Childcare centre staff will be provided with approximately two telephone calls by an HPO, pending childcare centre needs, within the intervention period [52, 53]. Barriers to centre implementation of healthy eating practices and use of Childcare EATS will be identified and strategies to address these barriers will be discussed. Email support will be provided by HPOs upon request by the centre. An additional training session delivered by an HPO via online modalities will be offered to centres, pending centre needs.

### Control group and contamination

Childcare centres allocated to the control group will receive usual care during the intervention period. Usual care includes general support from health promotion officer upon request to implement the state-wide obesity prevention program [27]. Support provided to centres within the HNE region to implement this state-wide program is centrally monitored by the research team. Enhanced support to implement the healthy eating practices targeted in the intervention will be offered to control centres after 12-month follow-up data collection is complete. Assessment of potential contamination will be collected via a telephone interview with nominated supervisors and centre champions during follow-up data collection.

### Outcomes

#### Feasibility of intervention

Feasibility of the intervention, defined as the extent to which the intervention can be successfully used or carried out within the childcare setting [54] for a fully-powered implementation trial, will be assessed through childcare centre and parent recruitment and consent rates for each component of data collection.

#### Childcare centre uptake of implementation strategies

Childcare centre use of Childcare EATS will be assessed through data provided via Google Analytics [55]. These analytics include, but not limited to, total time logged into the program, completion of the self-assessment and action plan, most frequently used program features and the number of requests for assistance. The research team has previously used these metrics to evaluate childcare centre adoption of web-based programs [21, 56]. Internal records detailing the provision of implementation strategies, including the completion, duration and centre staff in attendance at the educational outreach visit; type (i.e. telephone, email, online training), frequency and duration of ongoing support; centre staff signatories on the memorandum of understanding; and selection of a centre champion, will be maintained by research team members.

#### Acceptability of implementation strategies and intervention

Supervisors and champions of the childcare centres randomised to the intervention group will complete a telephone interview to assess acceptability during follow-up data collection (6 and 12 months). Acceptability will be defined as the perception amongst centre staff that the intervention and implementation strategies are agreeable, palatable or satisfactory [54]. This will be assess using modified items by Weiner et al. [57] and items previously used by the research team to capture data on perceived intervention effectiveness, unintended consequences, reach and adoption and acceptability (workforce, infrastructure, time requirements) [46, 58] and engagement with Childcare EATS [59].

#### Appropriateness of implementation strategies

Appropriateness, defined as the perceived fit, relevance or compatibility of the intervention and implementation strategies for the childcare setting [54], will be evaluated through information collected during follow-up telephone interviews with centre supervisors and champions. The telephone interviews will include modified items by Weiner et al. [57] and items used by the research team in previous childcare interventions [33].

#### Implementation context

Relevant constructs within three of the five domains of the CFIR [60] (inner setting (compatibility with centre values and direction, level of priority). innovation characteristics (perceived complexity and cost) and outer setting (external influences such as policies, regulations and peer behaviour)) will be used to identify factors associated with implementation at follow-up during a telephone interview with childcare supervisors [17, 60].

#### Potential effectiveness of the implementation strategy in improving implementation of targeted healthy eating practices

Childcare centre implementation of the targeted healthy eating practices (e.g. provision of intentional learning experiences about healthy eating and staff professional development in nutrition) and additional data on centre nutrition environments will be assessed with the Environmental and Policy Assessment and Observation (EPAO) tool [61]. Per EPAO protocol, a trained RA will undertake a one-day observation and review of childcare centre documentation. The EPAO has been previously used by the research team in 18 childcare centres [36] and has demonstrated high inter-observer agreement. The tool is considered to be gold standard for environmental observations in the childcare setting [61]. This will be undertaken at baseline and follow-up (6 and 12 months).

### Secondary effectiveness outcomes

#### Child dietary intake of fruit and vegetable servings in care

The mean servings of fruits and vegetables from all food and beverages consumed whilst in care will be assessed through the measurement of lunchbox foods and beverages across the day. On the days of the site visit, two trained RAs will assess the lunchboxes of participating children. Measurement of lunchbox contents will be conducted on two occasions across the day: prior to the children’s first meal time and after the children’s last meal time. RAs will remove all contents of the lunchbox and remove any lids that inhibit the view of contents. A photo will then be taken of the entire lunchbox contents. RAs will then weigh each food item included in the lunchbox, with strict adherence to safe food handling practices to address occupational health and safety concerns. A written description of the contents will also be captured to enable accurate recordings where ingredients may be easily deciphered via photograph, e.g. sandwiches and mixed meals. The process of photographing, weighing and recording lunchbox contents will be repeated after the children’s last meal. Consumption will be calculated based on foods and beverages present at the first measurement minus foods remaining at the second measurement. Educators will direct children to keep food wastage, including all partially consumed food and beverages, within their lunchboxes. All food wastage will be collected by the research team during the second measurement and factored into child consumption measurements. The weighed plate method with photographs has been previously used by the research team [36] and has proven to be a precise measure of dietary intake in previous studies [62]. This weighed food record data will be entered into a nutrient analysis database (FoodWorks) [63] by a trained dietitian blinded to centre allocation. During this process, the dietitian will categorise the food and beverage items into food groups and calculate mean servings of fruit and vegetables consumed in accordance with the serving sizes specified within the Australian Guide to Healthy Eating (AGHE) [64]. Photographs will be used to validate written descriptions of foods and the weights recorded. Lunchbox measurements will be conducted across the two-day site visits at three time points, baseline and follow-up (6 and 12 months).

#### Child dietary intake of sodium, saturated fat and added sugar in care

The nutrient output provided by weighed food record data entered into the nutrient analysis database (FoodWorks) following the process described above will be used to measure mean sodium (mg), saturated fat (g) and added sugars (g) from all foods and beverages consumed whilst in care.

#### Mean servings of fruit and vegetables packed within lunchboxes

To determine the impact of the intervention on parent provision of healthy food in lunchboxes, the mean servings of fruit and vegetables packed within children’s lunchboxes will be assessed via observation and measurement of lunchboxes and lunchbox foods and beverages following the same process described above.

#### Centre characteristics

Operational centre characteristics will be assessed at baseline during a telephone interview with nominated supervisors. Items within the telephone interview have been used previously by the research team [33], and include centre type (e.g. preschool, long day care), number of years in operation, days and hours of operation, postcode, number of children enrolled and attending, number of staff employed and the number of Aboriginal or Torres Strait Islander children enrolled at the centre.

#### Child characteristics

Child characteristics, including gender, age, Aboriginal and/or Torres Strait Islander origin, days attending care and parent level of education, will be collected from parents/guardians when providing written consent to participate in the study.

### Power calculations

As this is a pilot study, a formal sample size calculation for the primary outcome is not required [65]. However, we estimated the number of centres required as approximately 25% of the number needed for a fully-powered implementation trial. Based on consent rates from previous web-based intervention studies conducted within the ECEC setting and allowing for a childcare centre attrition rate at follow-up of 10%, it is estimated recruitment of 22 childcare centres would be sufficient to provide data to inform feasibility of undertaking the trial [21]. To assess the impact of the intervention on child diet, an approximate difference of 0.3 servings of both fruit and vegetables is considered clinically significant based on the potential reduction in risk of chronic disease [66, 67]. As such, given the 10% childcare centre attrition rate at follow up, recruitment of approximately 440 children from 22 childcare centres (20 children per centre) will enable detection of a mean difference of 0.3 servings in intake of fruit and vegetable servings, with an alpha of 0.05 and an estimated ICC of 0.1 [32], with 80% power [35, 68] and a standard deviation of 0.6 servings. Based on unpublished internal data, this number of participants will allow detection of a clinically meaningful difference of approximately 1.9 g saturated fat, 4.7 g added sugar and 155 mg sodium [69].

### Statistical analysis

The primary trial end-point will be the 6-month follow-up. Descriptive statistics will be used to describe childcare centre and child characteristics, the feasibility, uptake of implementation strategies, acceptability and appropriateness of the intervention and determinants of implementation. At the centre level, to determine the impact of the intervention on the implementation of healthy eating practices, scores of the EPAO will be compared between intervention and control centres at follow-up, adjusting for baseline, through linear regression analysis. At the child and centre levels, multiple imputations will be performed as part of a sensitivity analysis for missing follow-up data as recommended by White et al. [70]. At the child level, mixed linear regression models will be run on all secondary outcomes, where a group by time interaction will assess effectiveness of the intervention. All models will include a random effect for childcare centre to account for potential clustering effect, as well as fixed effects for prognostic variables (SES, gender) under an intention to treat framework. At the child level, subgroup analyses by centre socioeconomic status and child gender will also be undertaken to assess whether there was a differential impact of the intervention.

### Progression criteria

Data obtained from the trial will inform decisions regarding progression to a fully powered implementation trial. Such decisions will be made via majority, from core members of the research team, including a representative from a public health service partnering in the research that intends to adopt the intervention and implementation support strategy if identified as beneficial [71]. The decision will follow consideration and discussion between the core members of measures of feasibility, acceptability and appropriateness of the intervention and implementation strategies utilised in the study, and measures of the effect of the intervention on child dietary outcomes. Specifically, in order to progress, the team must deem the intervention, and implementation strategy to be sufficiently acceptable and feasible that it would likely be adopted by > 25% of childcare centres that were offered it. Or, that this could reasonably be expected with adaptations to the intervention or implementation approach based on steps previously employed by the research team [72]. Measures of implementation of the recommended practices, together with assessment of feasibility, uptake, acceptability and appropriateness will be used to identify opportunities to further strengthen its capacity prior to a fully powered implementation trial. This will enable the identification of implementation strategies and healthy eating policies and practices required to achieve the greatest outcome in implementation, and therefore, child diet.

## Discussion

Interventions targeting the childcare setting are recommended to improve child dietary intake in care due to the potential to reach a large number of children during a crucial developmental period [73]. Despite the existence of evidence-based healthy eating practice recommendations, previous findings on the impact of such recommendations on child diet in care are mixed [16]. Web-based interventions represent a promising modality to provide population-wide support to childcare centres given their potential reach and consistency with existing infrastructure [19]. This study will provide important data to support the conduct of a fully-powered implementation trial within Australian ECEC settings and inform the development of future implementation interventions.

## Supplementary information

**Additional file 1.** SPIRIT 2013 Checklist: Recommended items to address in a clinical trial protocol and related documents

**Additional file 2.** SPIRIT Figure

## Data Availability

Not applicable.

## Abbreviations

U.K.: United Kingdom
U.S.: United States
RCT: Randomised controlled trial
HNE: Hunter New England
NSW: New South Wales
RA: Research assistant
SES: Socioeconomic status
BCW: Behaviour Change Wheel
ERIC: Expert Recommendations for Implementing Change
HPO: Health promotion officer
EPAO: Environmental and Policy Assessment and Observation
CFIR: Consolidated Framework for Implementation Research
