# Supplementary material for: A pilot randomised controlled trial of a web-based implementation intervention to increase child intake of fruit and vegetables within childcare centres
Source: Pilot Feasibility Stud. 2020 Oct 29;6:163. doi: 10.1186/s40814-020-00707-w (PMC7597048; doi:10.1186/s40814-020-00707-w)
Supplement: Supplementary file 2 — Additional file 2. SPIRIT Figure [file 40814_2020_707_MOESM2_ESM.doc]

Figure 1: Schedule of enrolment, interventions, and assessments

|  | **STUDY PERIOD** | | | | |
| --- | --- | --- | --- | --- | --- |
|  | **Enrolment** | **Allocation** | **Post-allocation** | | **Close-out** |
| **TIMEPOINT** | ***-t_1_*** | **0** | ***0*** | ***6 months*** | ***12 months*** |
| **ENROLMENT:** |  |  |  |  |  |
| **Eligibility screen** | X |  |  |  |  |
| **Informed consent** | X |  |  |  |  |
| **Allocation** |  | X |  |  |  |
| **INTERVENTIONS:** |  |  |  |  |  |
| ***[Intervention]*** |  |  |  |  |  |
| **ASSESSMENTS:** |  |  |  |  |  |
| *[Child dietary intake of fruit and vegetable servings in care]* | X |  |  | X | X |
| *[Mean servings of fruit and vegetables packed within lunchboxes]* | X |  |  | X | X |
| *[Child dietary intake of sodium, saturated fat and added sugar in care]* | X |  |  | X | X |
| *[Childcare centre implementation of targeted healthy eating practices]* | X |  |  | X | X |
| *[Childcare centre uptake of implementation strategies]* |  |  |  | X |  |
| *[Feasibility of intervention and appropriateness of implementation strategies]* |  |  |  | X |  |
| *[Acceptability of implementation strategies and intervention]* |  |  |  | X |  |
| *[Implementation context]* |  |  |  | X |  |
